# Tuft Cells and Their Role in Intestinal Diseases

**DOI:** 10.3389/fimmu.2022.822867

**Published:** 2022-02-14

**Authors:** Sebastian Kjærgaard Hendel, Lauge Kellermann, Annika Hausmann, Niels Bindslev, Kim Bak Jensen, Ole Haagen Nielsen

**Affiliations:** ^1^ Department of Gastroenterology, Herlev Hospital, University of Copenhagen, Herlev, Denmark; ^2^ Novo Nordisk Foundation Center for Stem Cell Medicine, reNEW, Faculty of Health and Medical Sciences, University of Copenhagen, Copenhagen, Denmark; ^3^ Department of Biomedical Sciences , University of Copenhagen, Copenhagen, Denmark; ^4^ Biotech Research and Innovation Centre (BRIC), University of Copenhagen, Copenhagen, Denmark

**Keywords:** chemosensing, colorectal neoplasia, Crohn’s disease, inflammatory bowel disease, inflammation, intestine, tuft cells, ulcerative colitis

## Abstract

The interests in intestinal epithelial tuft cells, their basic physiology, involvement in immune responses and relevance for gut diseases, have increased dramatically over the last fifteen years. A key discovery in 2016 of their close connection to helminthic and protozoan infection has further spurred the exploration of these rare chemosensory epithelial cells. Although very sparse in number, tuft cells are now known as important sentinels in the gastrointestinal tract as they monitor intestinal content using succinate as well as sweet and bitter taste receptors. Upon stimulation, tuft cells secrete a broad palette of effector molecules, including interleukin-25, prostaglandin E_2_ and D_2_, cysteinyl leukotriene C_4_, acetylcholine, thymic stromal lymphopoietin, and β-endorphins, some of which with immunomodulatory functions. Tuft cells have proven indispensable in anti-helminthic and anti-protozoan immunity. Most studies on tuft cells are based on murine experiments using double cortin-like kinase 1 (DCLK1) as a marker, while human intestinal tuft cells can be identified by their expression of the cyclooxygenase-1 enzyme. So far, only few studies have examined tuft cells in humans and their relation to gut disease. Here, we present an updated view on intestinal epithelial tuft cells, their physiology, immunological hub function, and their involvement in human disease. We close with a discussion on how tuft cells may have potential therapeutic value in a clinical context.

## Highlights

1) Intestinal TCs form a central hub, involved in immune and regulatory metabolic networks, monitoring luminal intestinal content using chemosensory taste and succinate receptors, thus responding to a broad palette of substances and pathogens.

2) Upon stimulation TCs produce IL-25, ACh, TSLP, β-endorphins, and prostaglandins such as PGE_2_, PGD_2_ and LTC_4_ which are potent paracrine and endocrine signaling molecules, making them attractive potential therapeutic targets.

3) Identifying human intestinal TCs may require different markers from those used in murine experiments. These markers are still being investigated and evaluated. Suggested markers include COX-1, p-EGFR, SOX9, ALOX5, AVIL, girdin and ChAT, although none are TC specific.

4) Mouse studies have highlighted the importance of TCs in intestinal function, particularly in anti-helminthic and anti-protozoan immune responses, as well as in obesity models.

5) Certain human diseases such as inflammatory bowel disease, coeliac disease, and duodenal ulcer, are associated with alterations of intestinal TC populations and TC associated cytokines.

## Introduction

The intestinal epithelium serves a crucial role in maintaining gut mucosal homeostasis (1, 2). It forms a specialized physical and chemical barrier between self and non-self and controls activation of the host’s largest immune apparatus by interacting with triggers in the gut luminal content. At the same time, the epithelium is central for absorption of water, ions, and nutrients as well as secretion of ion-containing fluids with waste products especially in the colon.

The intestinal epithelial barrier is maintained by proliferating intestinal stem cells (ISCs) located at the bottom of the intestinal crypts. ISCs give rise to an array of differentiated cells scattered within the epithelial lining, including tuft cells (TCs) (3). While functions of most intestinal epithelial cells are well established, far less is known about the lately rediscovered TCs.

Despite their discovery more than sixty five years ago (4), a functional characterization of TCs was delayed into the 1990s due to lack of specific markers. Meanwhile, progress in TC-marker identification over the last 15 years has provided functional insights and identified more detailed hallmarks of TCs. These include their signaling pathways *via* luminal cues, apical receptors, second messengers and secretory mediators. Only recently a proximate consensus on detection of human TCs in the gut emerged, which now also enables clinical research on these cells. With this overview we aim to present A) what is currently known about the molecular basis of TC fate and differentiation as well as the identification of human intestinal TCs, and B) the available knowledge of involvement of TCs in diseases of the gastrointestinal (GI) tract, including a discussion of TCs in a clinical context and how elucidating TC functions and abundance might inspire novel therapeutic strategies.

## Tuft Cells

All segments of the GI tract, including esophagus, stomach, intestines, and associated organs, cooperate for nutrient uptake, and at the same time, they form the largest immune apparatus in the body and a physical barrier to the outside world containing a huge variety of potentially harmful agents. This special localization requires a tightly balanced immune surveillance achieved by specialized immune cells cooperating with non-immune cells such as epithelial cells, including TCs.

**Figure 1:**
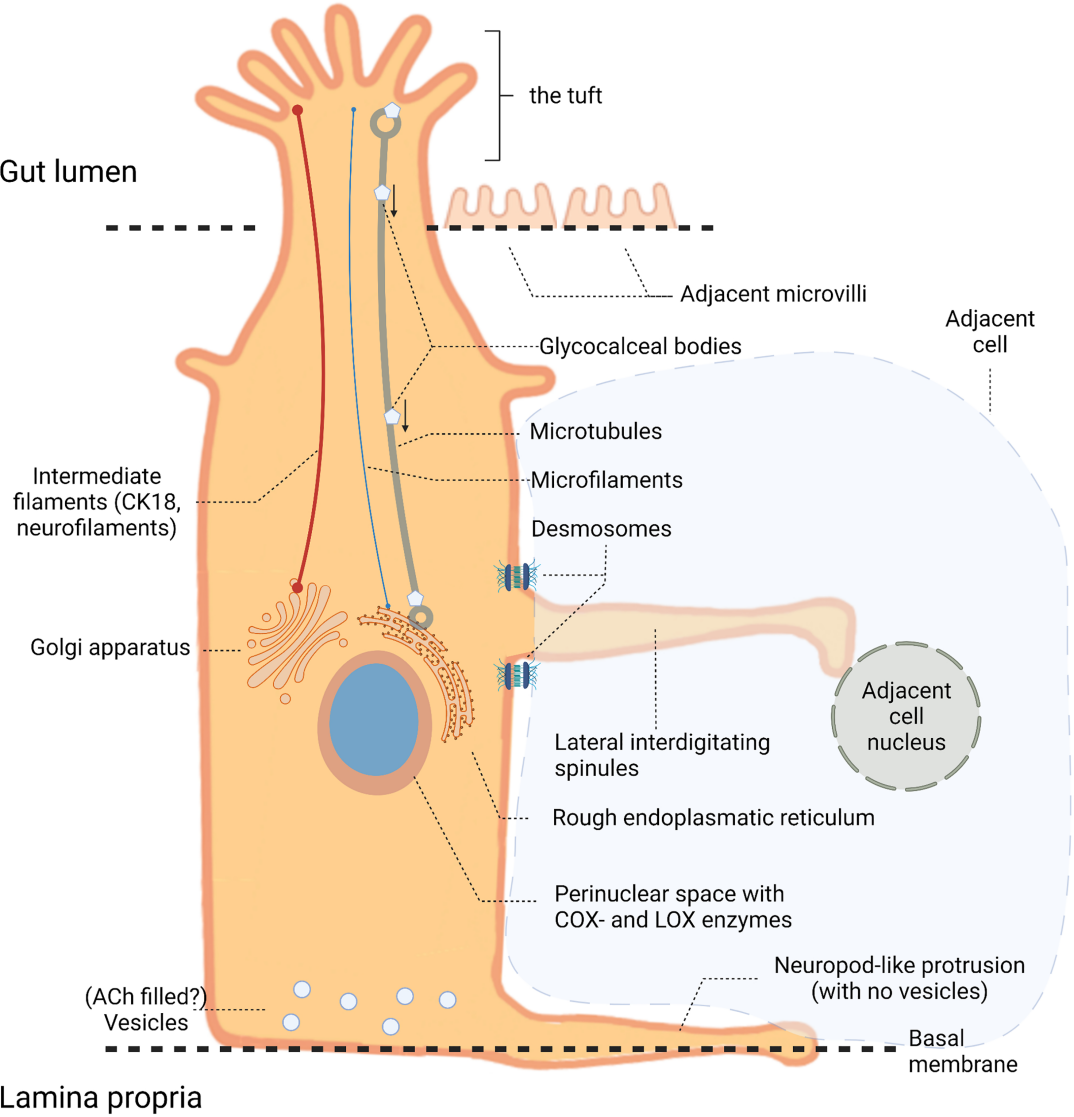
Tuft cell (TC) anatomy. Illustration of a TC located in the intestinal epithelium with its long apical microvilli (the “tuft”) extending into the gut lumen. Within the cell these microvilli associate with microtubules and microfilaments that extend towards the nucleus connecting to the perinuclear rough endoplasmic reticulum and Golgi apparatus. Intermediate filaments, such as cytokeratin-18 (CK18) and neurofilaments, contribute to the cytoskeletal make-up. At the nucleus level, cytoplasmic (or lateral interdigitating) spinules extend from the lateral TC border reaching the nuclei of neighboring cells. The basal part of TCs contains vesicles, unlike the neuropod-like protrusions extending towards the basal membrane.

TCs are found in many tissues, including airways (4), gallbladder (5), thymus (6), pancreas (7), urethra (8), and GI tract (9, 10), and have been described under various names such as brush-, solitary chemoreceptor-, microvillous-, fibrillovesicular- and caveolated cells. The name “tuft” is the latest addition to designators for this cell type and originates from its unique brush border morphology, **Figure 1**. TCs have a flask-shaped body with a narrow neck from which a tuft of microvilli extends from the apical membrane into the intestinal lumen (11). The microvilli are longer and more compact than those of neighboring non-TCs and are connected to a rich apical-basal oriented network of microfilaments and microtubules (5, 12). In TCs these microtubules extend deep down towards the nucleus connecting to the perinuclear endoplasmic reticulum. Membrane bound vesicles (glycocalceal bodies) have been identified both at the microvillus base and between microtubules, suggesting a function as a macromolecular-exchange route between the intestinal lumen and the endoplasmic reticulum, **Figure 1** (5, 11, 12). The cytoskeletal make-up further consists of intermediate filaments such as cytokeratin-18 (CK18) and neurofilaments, which are otherwise a distinctive feature of mature neurons (13). Some TCs have cytoplasmic spinules, which are projections from the lateral TC border that enter adjacent cells reaching their nuclei, and might function as a transport route for molecular cargo to and from adjacent cells. These projections appear on TCs ranging from just below the apical junctional complex to the nucleus level and are often associated with a pair of desmosomes at their base, **Figure 1** (5, 11). Also, the basal segments form neuropod-like protrusions extending along the basal lamina indicating paracrine signaling (11, 14, 15), although no secretory vesicles are observed within the TC neuropods. This is unlike the basal aspect of TCs close to adjacent neuronal fibers of the enteric nerve system, where vesicles are present presumably containing acetylcholine (ACh) for paracrine signaling (16).

Most of available data on TCs are based on studies in mice. The main part of this review therefore covers findings from mice, unless otherwise specified in the text.

## Intestinal TCs and Origin

The intestinal epithelium is particularly dynamic, as vigorously proliferating ISCs constantly renew the epithelium every 3-5 days (17). ISCs positive for leucine rich repeat containing G protein-coupled receptor 5 (Lgr5) are found at the crypt-bottom in both the small and large intestine and differentiate towards all the different epithelial lineages, including TCs (18, 19). Intestinal TCs are relatively rare as compared with other epithelial cell types. They are usually distributed in a sporadic and solitary manner, accounting for just 0.4-2.3% of the total epithelial cell population in murine intestinal epithelium (9, 20–22), while the only study on TC density of the human sigmoid colon report ~100 TCs per square millimeter tissue (23). Upon leaving the crypts, migrating along the villus axis, the function of TCs might change with increasing differentiation similar to what has recently been described for enteroendocrine cells (24–26). While the average TC has a turnover rate of 1-2 weeks (27), a small subpopulation of cells (~5%) expressing double cortin-like kinase 1 (DCLK1), a TC marker in mice, is remarkably long-lived, surviving for up to 18 months. These long-lived TCs may contribute to the epithelial regeneration following damage as they retain stem cell potential. Furthermore, they can give rise to organoids *in vitro* (small and large intestine), and might serve as cancer-initiating cells upon injury and deletion of adenomatous polyposis coli (APC) (28). Meanwhile, it remains uncertain whether these long-lived TCs represent a unique cell population, or a common secretory progenitor given that other secretory cells are shown to have similar properties (29).

### TC Differentiation

The identification of DCLK1 as a marker for TCs in mice facilitated their further characterization. It was quickly established that differentiation of ISCs to TCs is independent of the transcription factor neurogenin-3 (essential for differentiation of ISCs to intestinal enteroendocrine cells), and thus not associated with the enteroendocrine cell lineages (9). Instead, a common differential marker for all TCs across organs and regions is the taste-cell specific transcription factor, Pou2f3. Pou2f3 is a master regulator of and an absolute requisite for differentiation into TCs, indicating lineage similarity for lingual taste buds and TCs along the entire GI tract (30–33). Despite sharing this common transcription factor and being morphologically similar across tissues, transcriptional profiling using population-based and single-cell RNA sequencing (scRNA-seq) uncovered TC heterogeneity both across (34) and within tissues (22, 35–37). Haber et al. (22) highlighted two different clusters of TC progenitors, as well as two distinct clusters of mature TCs in the murine small intestine. One of them (denoted Tuft-1) expresses a neural development gene signature, whereas the other (denoted Tuft-2) is enriched with genes associated with immunological functions (22). Despite a major overlap in expression patterns between Tuft-1 and Tuft-2, solely Tuft-2 expresses thymic stromal lymphopoietin (TSLP) (22, 38), an epithelial derived cytokine promoting Th2 immunity. This suggests functional, and potentially spatial, heterogeneity of TCs (22). TC heterogeneity is further illustrated as an additional transcription factor associated with TC differentiation, Atonal bHLH transcription factor 1 (ATOH1), is region specific. ATOH1 is essential for the differentiation into secretory cell lineage (goblet cells, Paneth cells and enteroendocrine cells), as well as in the differentiation into TCs within the large intestine, while small intestinal TCs are ATOH1-independent (30, 39, 40). Krüppel-like factors such as Klf3 and Klf6 were also identified as transcription factors in small-intestinal TCs, while their roles in colonic TC development were not investigated (22). A recent study pinpointed the role of another intracellular signaling regulator in TC differentiation, namely sprouty2, which is mostly expressed in colonic epithelium compared to the remaining GI tract (41). It was demonstrated, both *in vitro* and *in vivo*, that acute colonic inflammation reduces sprouty2, leading to TC and goblet cell hyperplasia (41).

### Identification of Human Intestinal TCs

Identification of new reliable markers has provided insights into the function of TCs, including their involvement in diseases of the human body. However, the identification and characterization of TCs in the human intestinal tract based on marker expression is still awaiting consensus. Two studies (42, 43) describe a population of human colonic DCLK1^+^ TCs, however, Leppänen et al. (44) reported that human colonic epithelial DCLK^+^ cells have a morphology similar to absorptive enterocytes rather than a classical TC shape, thus questioning whether DCLK1 marks human TCs. In this context we recently tested a commercially available antibody (ab31704) on human colonic material, and failed to obtain convincing immunolabeling for DCLK1 (23), a conclusion also reached by Banerjee et al. (40). Differences in DCLK1 immunoreactivity in human GI epithelium could be due to differences in protocol and fixation methods or that human TCs simply do not express DCLK1. By contrast, cyclooxygenase enzyme (COX)-1 has been identified as a reliable marker for human TCs (9, 21, 23). Gerbe et al. (9) were first to validate COX-1 as a marker for human TCs by co-staining for other markers such as SRY-box transcription factor 9 (SOX9) and hematopoietic prostaglandin D synthase. Other useful markers include p-EGFR, arachidonate 5-lipoxygenase (ALOX5), advillin (AVIL), girdin and choline acetyltransferase (ChAT) (20, 21, 40, 45–47). Notably, none of these markers are TC specific. However, within the epithelium, they are often restricted to TCs, and a combination of one of these markers with a marker for epithelial cells, such as EpCAM or E-Cadherin, therefore faithfully identifies TCs (21).

### Tuft Cell Input

TCs monitor the intestinal lumen using a variety of apical receptors making them proficient sentinels of the GI tract as they respond to a broad palette of substances. Apical signaling includes taste receptors similar to those of taste buds of the tongue and soft palate. These receptors are usually divided into three types: Type 1 transduces signals for sweet and umami substances; type 2 for bitter substances, and type 3 registers sour substances (48). TCs express type 1 (Tas1Rs) and type 2 (Tas2Rs) (49–53). Another important receptor is succinate receptor 1, SUCNR1, which respond to succinate, a metabolite secreted by certain symbiotic bacteria, protists and helminths (54, 55). Receptor tyrosine kinases have also been identified in TCs, although the mechanism of activation is unclear. Basolateral signaling include other receptors, although not fully elucidated, such as gamma aminobutyric acid (GABA)-receptors in small intestinal TCs (55, 56), dopamine receptor Drd3 (22, 55), and the orphan adhesion G protein receptor ADGRG2 (26). Lastly, a recent study report a pathway for IgG activation of intestinal TCs, although only in a minor subpopulation of about 2.75% of TCs in the mouse small intestine (57).

#### Intestinal Chemosensing by Taste Receptors

Chemosensory taste receptors in the GI tract comprise G protein-coupled receptors of the two taste receptor families Tas1R and Tas2R. Several subtype receptors are expressed by different cell types all along the murine GI tract (58), and a varying expression pattern of these bitter taste subtype receptors are also identified along the human GI tract (48, 59–68). However, their region-specific expression distribution is not systematically corroborated by immunohistochemical studies.

**Figure 2:**
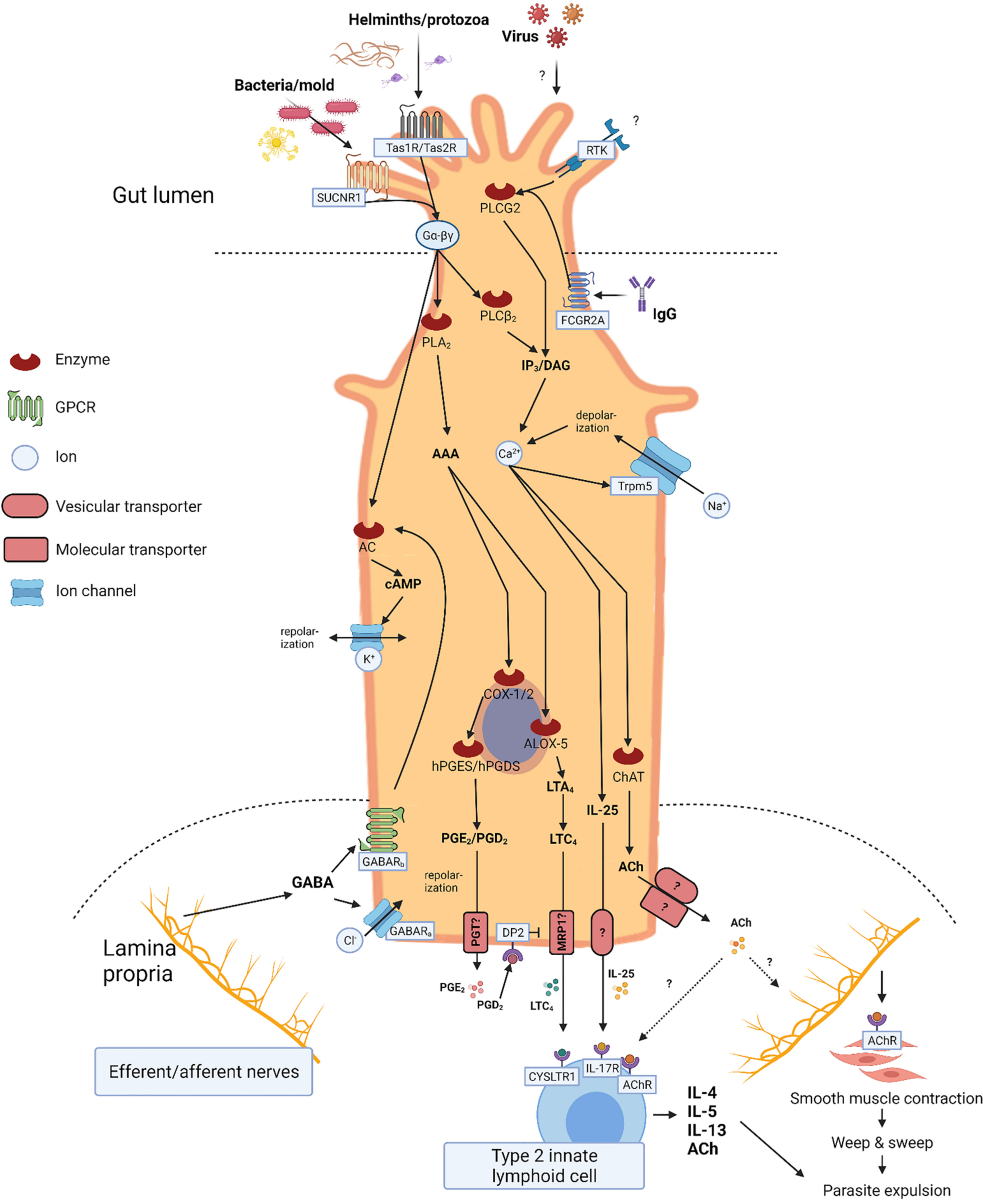
Tuft cell (TC) signaling. Illustration of TC signaling including input, output, and second messengers. Apical signaling involves luminal G-protein coupled receptors including succinate receptor 1 (SUCNR1), taste receptors (Tas1R/Tas2R), and receptor tyrosine kinases (RTK). Cell depolarization and IP3/DAG activity leads to increased intracellular calcium levels stimulating intracellular synthesis of effector molecules such as interleukin (IL)-25, acethylcholine (ACh), and eicosanoids. Activated arachidonic acid (AAA) is metabolized by cyclooxygenase enzyme (COX)-1/2, hematopoietic prostaglandin D synthase (hPGDS), and arachidonate 5-lipoxygenase (ALOX5) into prostaglandin E_2_ (PGE_2_), prostaglandin D_2_ (PGD_2_), and cysteinyl leukotriene C_4_ (LTC_4_), respectively. Basolateral secretion of IL-25 and ACh occurs through yet unknown mechanisms, whereas secretion of PGE_2_ and PGD_2_ is facilitated through the prostaglandin transporter (PGT), and LTC_4_ possibly through the multidrug resistance protein 1 (MRP1). Basolateral signaling involves GABAA (ligand-gated chloride channel) and GABAB (G-protein coupled) receptors. Stimulation initiates cell repolarization mediated by GABA receptors.

The first indication of chemosensory functions of TCs was reported in 1996. Here, Höfer et al. (69) identified brush cells of the gut expressing α-gustducin (a G-protein subunit involved in sweet, umami, and bitter taste signal transduction), which was thought to be specific for taste receptor cells of the tongue. Recent reports indicate the presence of Tas1R and Tas2R taste receptors in TCs of the GI tract (49–53) as well as of the complete set of down-stream entities in the taste receptor signaling cascade. Out of these, the monovalent cation conductive and calcium-regulated Trpm5 channel is specific for the TCs (although not specific for taste signaling transduction) in the GI epithelium, **Figure 2** (32, 38, 48, 50, 68–70). Although the mechanism has not been fully established, TCs use taste receptors to identify helminths and protozoa. For instance, the Tas1R3 in mice is central in TC activation by the protozoa *Tritrichomonas muris*, but not by the helminth *Heligmosomoides polygyrus* (49). On the contrary, *Heligmosomoides polygyrus* appear to actually *inhibit* tuft and goblet cell gene expression and expansion (71). Furthermore, in an experimental model with small intestinal organoids from mice, Luo et al. (51) observed calcium responses and interleukin (IL)-25 secretion from TCs when stimulating with excretory/secretory products, and extract from the helminth *Trichinella spiralis* as well as the bitter compound salicin – a response mediated through Tas2Rs (sensing bitter substances) expressed by TCs. These responses were blocked by the bitter-taste receptor inhibitor allyl isothiocyanate. Similarly, denatonium, another bitter substance, elicits a strong increase in intracellular calcium levels in *ex vivo* stimulated colonic TCs (72). However, more studies are required to determine the role of taste receptors in TC activation.

#### Succinate Receptor 1, SUCNR1

Succinate, a microbial metabolite, stimulates IL-25 secretion from TCs *via* SUCNR1, thus triggering a type 2 immune response, including activation of type 2 innate lymphoid cells (ILC2), eosinophilia and differentiation of tuft- and goblet cells (54). The highest TC expression of SUCNR1 has been observed in ileal TCs, although some SUCNR1 expression was also observed in colonic samples (73).

#### Gamma Aminobutyric Acid (GABA)-Receptors

The function of GABA is best characterized in the central nervous system but is also expressed in other tissues such as the GI tract. Although the role of GABA signaling in GI function (e.g., motility, blood flow, secretion, and immune cell activation) is not fully understood, it is well established that neurons of the myenteric plexus and mucosal endocrine-like cells synthesize and secrete GABA (74). Studies examining the role of GABA in modulating immune function indicates both protective and aggravating properties of GABA signaling, suggesting a complex signaling pattern (75, 76). TCs were shown to express both GABA_A_ and GABA_B_ receptors (55, 56), although the significance of this has yet to be elucidated.

### Tuft Cell Network Signaling

TCs are first responders to a range of luminal stimuli and diligently communicate with the mucosal immune apparatus and neuronal network. Most TCs express an enzymatic apparatus enabling them to synthesize a variety of paracrine and endocrine effector molecules, including IL-25 (32, 38, 50), ACh (21), eicosanoids (55, 77), β-endorphins (9), and TSLP (22) (**Figure 2**).

#### IL-25

TCs represent a major epithelial source of IL-25, a critical cytokine for the initiation of type 2 immune responses against helminths and protozoan parasites - and potentially other enteric pathogens - as part of the small intestinal immune system (32, 38, 40, 50, 54, 78). During homeostasis, TCs secrete small amounts of IL-25, while secretion is substantially increased upon activation, e.g., during helminth infection. A subsequent rapid IL-25 mediated expansion of ILC2s provides a crucial early source of IL-13, which in turn stimulates differentiation of intestinal stem cells toward a tuft- and goblet cell fate, and is key to the intestinal “weep and sweep” response expelling intruding parasites (32, 38). ILC2s also secrete IL-4, IL-5 and ACh, all contributing to parasite clearance (32, 79, 80). Several other immune cells of the lamina propria, such as natural killer T cells (81) and nuocytes (an innate type 2 immune effector leukocyte) (82), also play a role in the IL-25 anti-helminth response circuit. Their contributions remain to be fully elucidated, unlike TCs, that are proven indispensable for mounting a proper anti-helminth response (32).

#### Eicosanoids

Expression of COX-1 and -2 as well as 5-lipoxygenase enables TCs to synthesize prostaglandin D_2_ (PGD_2_) and -E_2_ (PGE_2_) and cysteinyl leukotriene C_4_ (LTC_4_) (55), with important physiological roles both during homeostasis (e.g., GI motility, secretion, and mucosal protection) as well as under pathological conditions (e.g., inflammatory bowel disease (IBD), and colorectal cancer) (83, 84). Specifically, intestinal inflammation has been associated with increased COX-2 enzyme activity and PGE_2_ production (85, 86), although PGE_2_ can exert either pro- or anti-inflammatory effects depending on the context (87). LTC_4_ is a further potent inflammatory mediator, synthesized *via* the lipoxygenase pathway when stimulated by helminths or protozoa through a yet unknown receptor mechanism (77, 88, 89). While LTC_4_ works synergistically with IL-25, it is strikingly dispensable for anti-helminth immunity. This is in surprising contrast to IL-25, which is essential to worm-expulsion. Interestingly, both the synthesis and degradation of LTC_4_ are more rapid than that of IL-25 (77, 90).

#### Acetylcholine

ACh, the main neurotransmitter in cholinergic neurons, is also an important modulator of epithelial proliferation and intestinal physiology (91). In the human GI tract, TCs are the only epithelial cell type expressing ChAT, the enzyme required for ACh production (21). It is still unclear what stimulates ACh synthesis in TCs, but it is likely a result of activation of the canonical taste receptor signaling pathway (92, 93). Recently, an important and thorough study was published on possible debris products of bacterial origin as stimuli for ACh-dependent auto- and paracrine pathways in chemosensory cells specific for activation of tracheal clearance in mice. Conclusions in the study are certainly relevant for TC function in the intestine (94). Their study shows that a cluster of three subtype bitter taste receptors (-126, -135 and -143), when eliminated, results in a significant 44% reduction of an ACh-induced peak effect for tracheal clearance. The same was true for contraction induced in urinary bladder strips by a significant 30% reduction. The tracheal chemosensory cells express 18 subtypes of which some, but not all, could be discarded as involved in the remaining ACh-signaling. Hence, more studies on this subject, also for intestinal TCs, are needed before a firm conclusion can assess additional involvement of bitter taste receptors on downstream ACh-induction as well as in general.

In neurons, vesicular acetylcholine transporter (VAChT) is responsible for loading ACh into secretory organelles for secretion. According to Schütz et al. (72), murine intestinal TCs do not express VAChT except for few TCs in the ascending (proximal) colon. VAChT immunoreactive epithelial cells of the human sigmoid (distal) colon, however, have previously been described (95). As such, the release of ACh from TCs is still not clarified and may involve different secretion mechanisms compared to those of neurons. For instance, transporters that can export ACh from colonic epithelium cells in a non-vesicular fashion have been described in human sigmoidal epithelium as well as choline importers and several enzymes needed for ACh synthesis. Future work should address potential co-localization of these transporters with TCs (96).

#### β-Endorphin and Thymic Stromal Lymphopoietin

Intestinal TCs also secrete β-endorphins and thymic stromal lymphopoietin, although the importance of these TC derived effector molecules is unclear (22). For instance, opioids, such as β-endorphin, are usually associated with analgesic effects, but are also involved in basic GI physiology including intestinal motility, secretion, and absorption (97). In the GI tract, expression of β-endorphins is restricted to TCs (9, 98). Notably, all intestinal TCs produce β-endorphins (9), and the secretion hereof depends on the calcium-regulated Trpm5 channel, which is specific for TCs within the intestinal epithelium. TSLP, on the other hand, is a cytokine promoting Th2 immunity similar to IL-25 (99). Some basolateral TSLP secretion derives from TCs (38), and secretion is increased upon tissue damage and exposure to pathogens (100, 101). Of note, TSLP appears to be involved in various immunological conditions including IBD (102).

## Tuft Cells and Gastrointestinal Diseases

### Mouse Obesity as a Chronic Low-Level Inflammation Model

Obesity is a condition with gradually increasing prevalence worldwide, representing a risk factor for developing a variety of diseases including several GI disorders and changes in GI motility. Recently, the effects of high fat diet and induced obesity on small intestinal TCs was examined in a mouse model. Although the ratio of number of TCs to total epithelial cells was unaltered, TC specific expression of IL-25 and TLSP was decreased (56). Altered GABA(A) and GABA(B) receptor activation pathways correlated positively with the altered expression of TC signature genes IL-25 and TSLP, suggesting an association between TC numbers and GABA receptor signaling (56).

### Infections of the GI Tract

An essential role of TCs in anti-helminthic and anti-protozoan immunity has been established. TCs respond to such parasites by secreting IL-25 and LTC_4_ initiating a type 2 immune response ultimately expelling the parasitic agent (32, 38, 50, 77). Generally, immunomodulatory effects and potentially beneficial effects of parasitic worms on intestinal function, inflammation and mucosal healing have been studied in detail in mice and humans (103). Considering the role of TCs in anti-helminthic/protozoan immunity it is tempting to speculate that TCs could mediate these effects. However, only sparse data exists on humans, and so far, helminth therapy appears to have no obvious effect compared to placebo treatment in the management of human disorders like IBD (104, 105). Furthermore, helminth therapy has its drawbacks given that prolonged exposure may cause complications as the host is exposed to a wide spectrum of helminth-derived products, including potent antigens and inflammatory stimuli, in addition to desired helminth immunomodulators. Although short-term administration of *Trichuis suis ovae* (pig worm eggs) appears to be safe and tolerable in humans (104, 106, 107), individuals exposed to chronic helminth infections appear to develop an immune dysregulation. This dysregulation results in hypo-responsiveness and difficulties in mounting proper immune responses, e.g. towards neurotropic flaviviruses, an effect attributed to the persistent immune activation (108, 109). The effect is thought to be mediated by TCs *via* an IL-4 signaling pathway resulting in a compromised CD8^+^ T cell response (109).

TCs also appear to be a target of norovirus, which is the leading cause of gastroenteritis worldwide. In mice, TCs express high levels of murine norovirus receptors, CD300lf, enabling infection, while also serving as viral reservoirs causing chronic infections and viral shedding for weeks following the acute phase of infection (110, 111).

Yet another candidate driver of TC induced immune responses is succinate, which indirectly activates the host immune system through SUCNR1 receptors also expressed on TCs (54). Succinate, a metabolite produced by commensal bacteria and helminths, has been shown to induce ATOH1-independent (small intestinal) TC expansion. In line with the protective TC functions described above, administration of succinate in two mouse models suppressed ileal inflammation and restored epithelial architecture, accompanied by an enhanced anti-parasite-, and a reduced type Th17 response (40). However, no increase in TC numbers following oral succinate administration was observed in the cecum and colon of mice, limiting the succinate response *via* TCs to the small intestine (54). How increased luminal succinate levels link to cytokine expression and downstream responses remains to be shown. Interestingly, vancomycin-induced dysbiosis (potentially leading to colonization of *Clostridioides (C). difficile*) has been associated with TC hyperplasia. Although a direct involvement of TCs in *C. difficile* infections has so far not been studied, neither in mice nor in humans, some studies indicate a correlation (112). In *C. difficile* pathophysiology IL-23 is considered mainly responsible for the destructive inflammatory response leading to severe tissue damage (113, 114), whereas IL-25, the main TC derived cytokine, demonstrates protective properties (115). The effect is thought to be mediated through IL-25 stimulated IL-13 release and recruitment of eosinophils; a hallmark of type 2 immunity and a TC characteristic (116).

### Inflammatory Bowel Disease

IBD is a group of chronic idiopathic inflammatory disorders characterized by latent symptom-free periods and recurrent flares with varying degrees of inflammation of the GI tract (117–119). The two most prevailing entities are ulcerative colitis (UC) and Crohn’s disease (CD). Symptoms such as abdominal discomfort, urge to defecate, (bloody) diarrhea and fatigue may be present in both disorders, they differ greatly in terms of pathophysiology although the pathogenesis is still not fully elucidated (119).

Accumulating evidence has demonstrated that T helper cell (T_h_)1-related cytokines (e.g., tumor necrosis factor-α (TNF-α), interferon-γ and IL-12) as well as T_h_17-associated cytokines like IL-17A, IL-21 and IL-23 are markedly increased in the inflamed mucosa of patients with CD, whereas the cytokine profiles in inflamed mucosal areas in patients with UC seem to exhibit increased production of T_h_2 associated cytokines, including IL-4, IL-5, and IL-13 (119, 120). By contrast, Su et al. (121) found IL-25 to be significantly decreased in both serum and inflamed intestinal mucosa of patients with flaring IBD compared to healthy controls. The same trend was observed in non-inflamed tissue and serum from patients with quiescent UC and CD. After treatment with infliximab (a TNF-α inhibitor) for flaring of IBD, serum IL-25 levels were, however, restored (121).

Thus, a critical role of IL-25 has been suggested in the pathogenesis of IBD, and accordingly increasing IL-25 levels could be a potential therapeutic option for IBD. As TCs are potent producers of IL-25 it is tempting to speculate about a potential role in IBD pathophysiology. However, the above-mentioned study by Su et al. (121) did not specifically investigate the role of TCs, nor did it differentiate between epithelial and subepithelial IL-25 expression. Several immune cells of the lamina propria produce and secrete IL-25, and TCs alone might not fully account for the observed changes (121, 122). Banerjee et al. (40) did observe a reduced TC population in inflamed ileal tissue in CD, which is similar to what was demonstrated in the non-inflamed colon of patients with UC in clinical, endoscopic and histologic remission (23). Whether this reduction in TC numbers in both UC and CD is a consequence of the disease or an actual contributing pathogenic factor remains unclear.

### Coeliac Disease, Duodenal Ulcer and Acute Duodenitis

A recent study by Huh et al. (47) described the TC abundance in biopsy specimens from pediatric patients with acute and chronic duodenitis. TC numbers were lower in patients diagnosed with coeliac disease and duodenal ulcers when compared to healthy specimens. In acute duodenitis, TC numbers decreased as the severity of inflammation increased, indicating a loss of TC differentiation or death during active inflammation. Whether inflammation precedes TC decimation or is a consequence hereof is still unclear.

### Gastric and Colorectal Neoplasia

**Figure 3:**
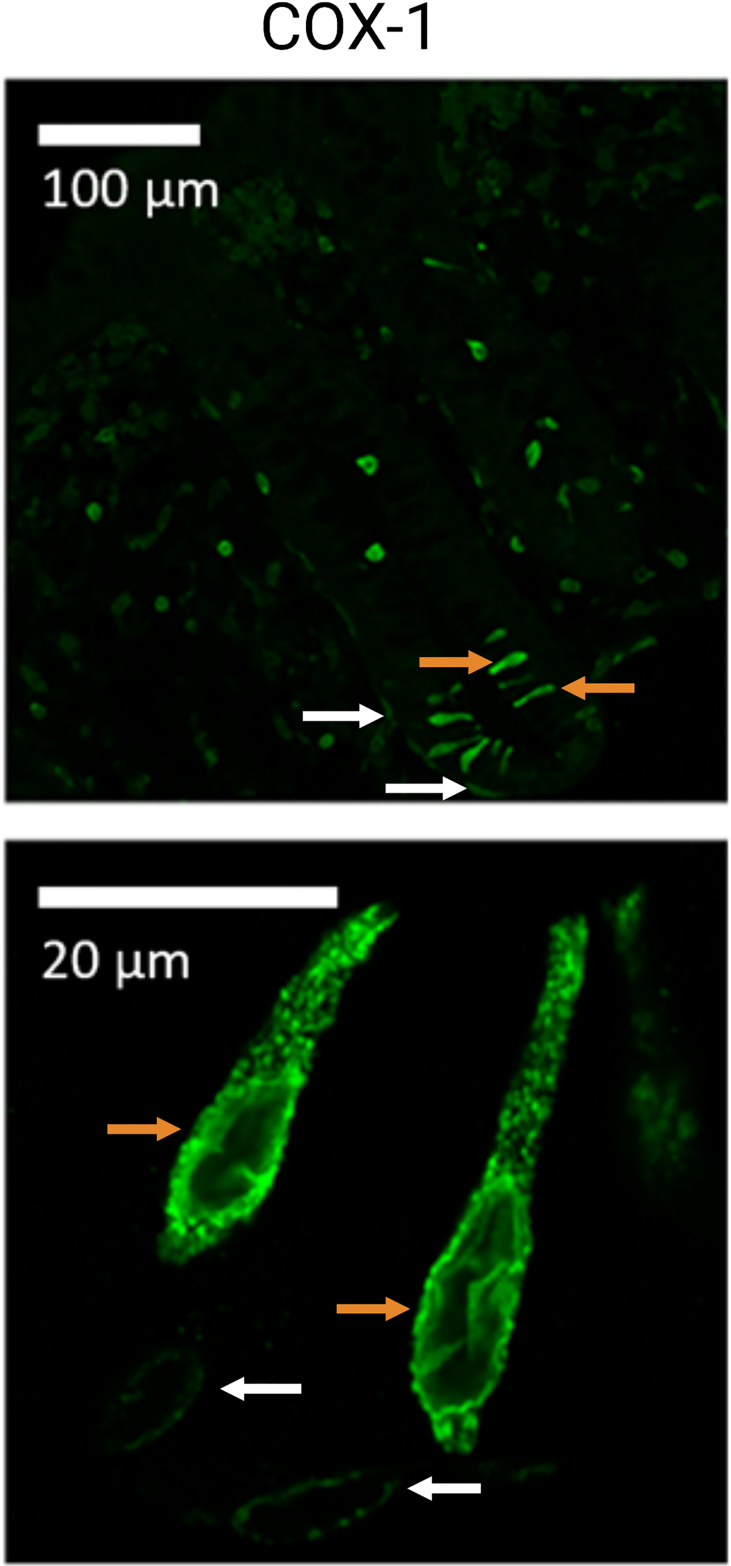
Cyclooxygenase 1 (COX-1) positive tuft cells (TC) of the human colon. Illustration of colonic TCs from healthy human sigmoid biopsies (controls). Orange arrows indicate TCs, while white arrows indicate what could be interpreted as subepithelial/pericryptal fibroblasts. Adapted from “*Possible predisposition for colorectal carcinogenesis due to altered gene expressions in normal appearing mucosa from patients with colorectal neoplasia*” by Petersen et al. (126).

TCs are massively expressed in locations of the ventricle, including epithelial areas of the cardia-near esophagus, where they seem to protect and repair the epithelium, for instance after acidic injury by gastro-esophageal reflux ultimately leading to Barrett’s adenocarcinoma (123). Recent insights kindle speculations about the genesis of sporadic intestinal tumors where TCs might be involved in the tumor etiology, although a direct involvement of TCs in the development of colorectal neoplasia (CRN) has not been reported. In this context, Roulis et al. (124) found rare pericryptal fibroblasts expressing COX-2, located in close proximity of the stem cell zone, that stimulated the expression of markers associated with regeneration (Sca-1+ cells) and thus demonstrated possible tumor-initiation properties *via* paracrine PGE_2_–signaling (124, 125). Similarly to TCs, these mesenchymal cells expressed both COX-1 and COX-2. In human colonic mucosa, COX-1 positive TCs are identified located within the epithelium together with subepithelial COX-1 expressing cells that may represent the pericryptal fibroblasts, **Figure 3** (126). Both the TCs and subepithelial cell types are located near the stem cell zone. As TCs seem to be a major source of prostaglandins they may also be a contributing cell type to CRN development (9). Indeed, an imbalance of arachidonic acid metabolism, including prostaglandin synthesis as well as production of free oxygen radicals, is suspected to be central to the pathogenesis of CRN (83, 127).

## Discussion

The number of studies examining TCs is increasing rapidly. Several breakthroughs elucidating TC function and significance have recently been made, including the identification of TCs as first responders to intestinal parasitic infections. Despite their relatively low numbers, TCs are important sentinels of the GI tract monitoring the luminal content with an array of luminal receptors responding to a broad palette of substances such as succinate, canonical tastes (sweet, bitter, umami) and secretory agents from parasitic helminths and protozoa. A close physical and functional relation to enteroendocrine cells and the underlying immune cells and nervous apparatus enable TCs to mediate responses to metabolic shifts in the intestinal lumen expelling potentially harmful agents such as parasitic worms.

However, our knowledge of TCs is still limited, especially concerning their involvement in human diseases. Observations from murine studies suggest an involvement of TCs in multiple diseases, e.g., neoplasia and colitis, but only few studies deal with human TCs, partly due to a late identification of relevant markers of human TCs. These are still being investigated, while possible markers, although not TC specific, include COX-1, p-EGFR, SOX9, ALOX5, AVIL, girdin and ChAT.

It appears that the number of intestinal TCs change during human pathologies. Whether this is merely a consequence of the disease or an actual contributing pathogenic factor, is a question that requires further exploration. With the help of new, specific markers and an improved understanding of TC heterogeneity, it is of importance to outline their potential as future diagnostic/predictive markers and to understand potential therapeutic benefits of modulating TC abundancy.

**Figure 4:**
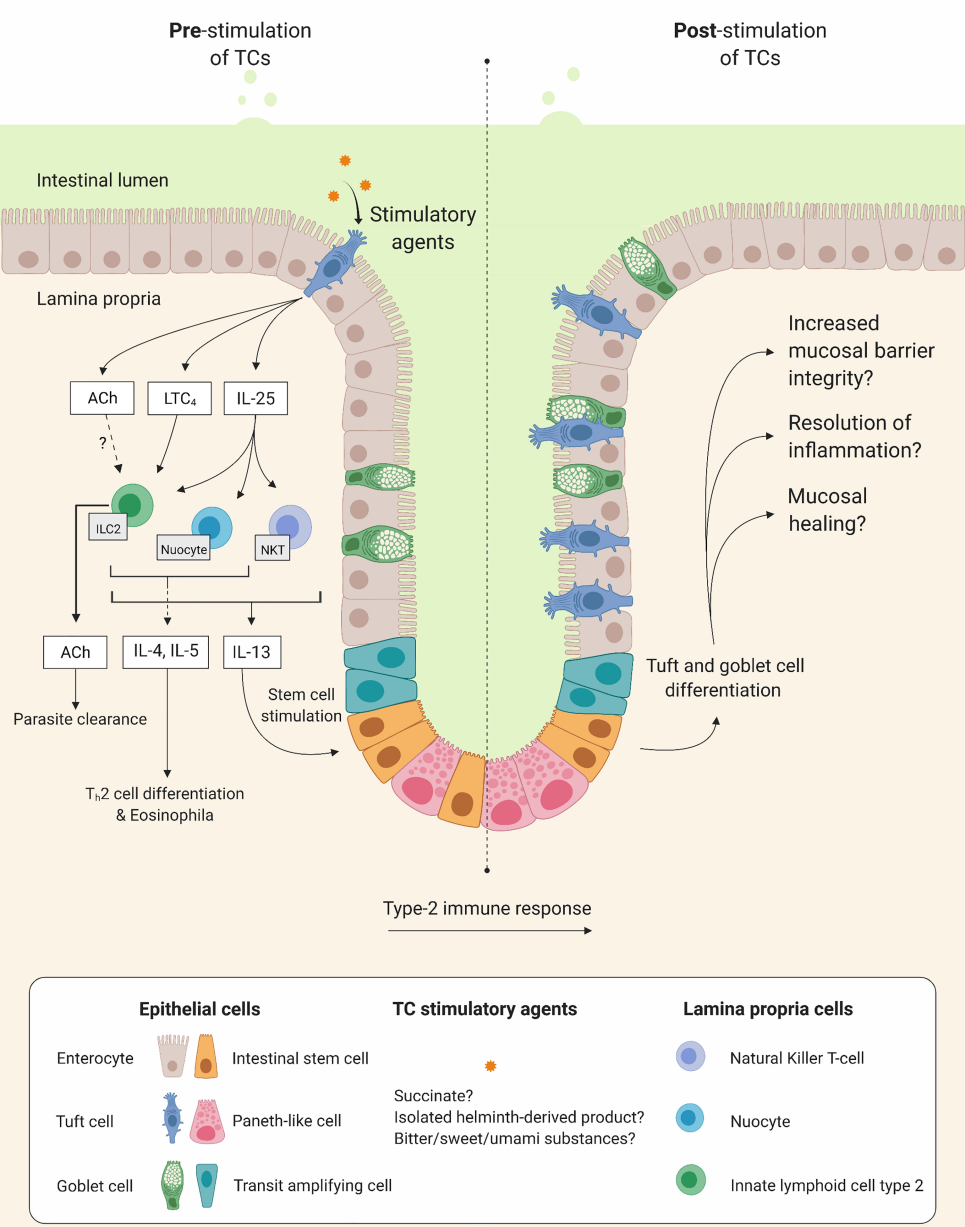
Tuft cells (TC) as potential therapeutic targets. Illustration of hypothetical beneficial effects on mucosal integrity when stimulating TCs. Left and right side show intestinal mucosa before and after TC stimulation, respectively. Upon stimulation, TCs increase secretion of interleukin (IL)-25 and cysteinyl leukotriene C_4_ (LTC_4_) which in turn stimulate lamina propria immune cells such as, type 2 innate lymphoid cells (ILC2), natural killer T cells and nuocytes to produce and release type 2 inflammatory cytokines (e.g. IL-4, IL-5 and IL-13). This initiates differentiation of T helper type 2 cells (T_h_2), recruitment of eosinophils and stimulation of intestinal stem cells leading to tuft cells and goblet cell differentiation and hyperplasia.

It is tempting to speculate that stimulating TC expansion in parallel with other cell populations, such as goblet cells, ILC2s and eosinophils, could promote epithelial barrier integrity and homeostasis and have a preventive effect on disease progression and risk of/severity at a new flare. TC specific activation may be achieved by substances activating canonical taste receptor signaling pathways, e.g., certain sweet, bitter, and umami substances, or even parasitic derived products. The isolation and administration of certain helminth derived products could have a beneficial effect and circumvent the negative consequences of chronic infections with helminths or other parasites. An illustration of the hypothetical beneficial effects of TC stimulation is shown in **Figure 4**.

Accordingly, a better understanding of TC biology may ultimately contribute to the efforts of prevention and development of novel treatment strategies against a wide range of human disorders of the GI tract.

## Author Contributions

This study was conceived by SH and ON. SH wrote 1^st^ draft. LK, AH, NB, KJ, and ON critically edited and approved the manuscript. All authors contributed to the article and approved the submitted version.

## Conflict of Interest

The authors declare that the research was conducted in the absence of any commercial or financial relationships that could be construed as a potential conflict of interest.

## Publisher’s Note

All claims expressed in this article are solely those of the authors and do not necessarily represent those of their affiliated organizations, or those of the publisher, the editors and the reviewers. Any product that may be evaluated in this article, or claim that may be made by its manufacturer, is not guaranteed or endorsed by the publisher.

## Abbreviations

ACh, Acetylcholine; ALOX5, Arachidonate 5-lipoxygenase; ATOH1, Atonal bHLH transcription factor 1; AVIL, Advillin; C. difficile, Clostridioides difficile; CD, Crohn’s disease; ChAT, Choline acetyltransferase; CK18, Cytokeratin-18; COX, cyclooxygenase enzyme; CRN, Colorectal neoplasia; DCLK1, Double cortin-like kinase 1; GABA, Gamma aminobutyric acid; GI, Gastrointestinal; IBD, Inflammatory bowel disease; ISC, Intestinal stem cell; IL, Interleukin; ILC2, Type 2 innate lymphoid cell; LTC_4_, Cysteinyl leukotriene C_4_; PGD_2_, Prostaglandin D_2_; PGE_2_, Prostaglandin E_2_; scRNA-seq, Single-cell RNA sequencing; SOX9, SRY-box transcription factor 9; SUCNR1, Succinate receptor 1; TasR, taste receptor; TC, Tuft cell; TSLP, Thymic stromal lymphopoietin; UC, Ulcerative colitis; VAChT, Vesicular acetylcholine transporter.

## References

[1] Peterson LW Artis D. Intestinal Epithelial Cells: Regulators of Barrier Function and Immune Homeostasis. Nat Rev Immunol (2014) 14:141–53. doi: 10.1038/nri3608 24566914

[2] Allaire JM Crowley SM Law HT Chang SY Ko HJ Vallance BA. The Intestinal Epithelium: Central Coordinator of Mucosal Immunity. Trends Immunol (2018) 39:677–96. doi: 10.1016/j.it.2018.04.002 29716793

[3] Larsen HL Jensen KB. Reprogramming Cellular Identity During Intestinal Regeneration. Curr Opin Genet Dev (2021) 70:40–7. doi: 10.1016/j.gde.2021.05.005 34062491

[4] Rhodin J Dalhamn T. Electron Microscopy of the Tracheal Ciliated Mucosa in Rat. Z für Zellforsch und Mikroskopische Anat (1956) 44:345–412. doi: 10.1007/BF00345847 13353477

[5] Luciano L Reale E. A New Morphological Aspect of the Brush Cells of the Mouse Gallbladder Epithelium. Cell Tissue Res (1979) 201:37–44. doi: 10.1007/BF00238045 527013

[6] Panneck AR Rafiq A Schütz B Soultanova A Deckmann K Chubanov V. Cholinergic Epithelial Cell With Chemosensory Traits in Murine Thymic Medulla. Cell Tissue Res (2014) 358:737–48. doi: 10.1007/s00441-014-2002-x PMC4233111 25300645

[7] DelGiorno KE Naeem RF Fang L Chung CY Ramos C Luhtala N. Tuft Cell Formation Reflects Epithelial Plasticity in Pancreatic Injury: Implications for Modeling Human Pancreatitis. Front Physiol (2020) 11:88. doi: 10.3389/fphys.2020.00088 32116793 PMC7033634

[8] Perniss A Schmidt P Soultanova A Papadakis T Dahlke K Voigt A. Development of Epithelial Cholinergic Chemosensory Cells of the Urethra and Trachea of Mice. Cell Tissue Res (2021) 385:1–15. doi: 10.1007/s00441-021-03424-9 33616728 PMC8270884

[9] Gerbe F Van Es JH Makrini L Brulin B Mellitzer G Robine S. Distinct ATOH1 and Neurog3 Requirements Define Tuft Cells as a New Secretory Cell Type in the Intestinal Epithelium. J Cell Biol (2011) 192:767–80. doi: 10.1083/jcb.201010127 PMC3051826 21383077

[10] Saqui-Salces M Keeley TM Grosse AS Qiao XT El-Zaatari M Gumucio DL. Gastric Tuft Cells Express DCLK1 and Are Expanded in Hyperplasia. Histochem Cell Biol (2011) 136:191–204. doi: 10.1007/s00418-011-0831-1 21688022 PMC3570962

[11] Hoover B Baena V Kaelberer MM Getaneh F Chinchilla S Bohórquez DV. The Intestinal Tuft Cell Nanostructure in 3D. Sci Rep (2017) 7:1652. doi: 10.1038/s41598-017-01520-x 28490731 PMC5431925

[12] Luciano L Reale E. Brush Cells of the Mouse Gallbladder - A Correlative Light- and Electron-Microscopical Study. Cell Tissue Res (1990) 262:339–49. doi: 10.1007/BF00309889 2076538

[13] Luciano L Groos S Reale E. Brush Cells of Rodent Gallbladder and Stomach Epithelia Express Neurofilaments. J Histochem Cytochem (2003) 51:187–98. doi: 10.1177/002215540305100207 12533527

[14] Cheng X Voss U Ekblad E. Tuft Cells: Distribution and Connections With Nerves and Endocrine Cells in Mouse Intestine. Exp Cell Res (2018) 369:105–11. doi: 10.1016/j.yexcr.2018.05.011 29758188

[15] Cheng X Voss U Ekblad E. A Novel Serotonin-Containing Tuft Cell Subpopulation in Mouse Intestine. Cell Tissue Res (2019) 376:189–97. doi: 10.1007/s00441-018-02988-3 30666535

[16] Middelhoff M Westphalen CB Hayakawa Y Yan KS Gershon MD Wang TC. Dclk1-Expressing Tuft Cells: Critical Modulators of the Intestinal Niche? Am J Physiol - Gastrointest Liver Physiol (2017) 313:G285–99. doi: 10.1152/ajpgi.00073.2017 PMC5668570 28684459

[17] Darwich AS Aslam U Ashcroft DM Rostami-Hodjegan A. Meta-Analysis of the Turnover of Intestinal Epithelia in Preclinical Animal Species and Humans. Drug Metab Dispos (2014) 42:2016–22. doi: 10.1124/dmd.114.058404 25233858

[18] Barker N Van EsJH Kuipers J Kujala P Den BMV Cozijnsen M. Identification of Stem Cells in Small Intestine and Colon by Marker Gene Lgr5. Nature (2007) 449:1003–7. doi: 10.1038/nature06196 17934449

[19] Clevers H. The Intestinal Crypt, a Prototype Stem Cell Compartment. Cell (2013) 154:274–84. doi: 10.1016/j.cell.2013.07.004 23870119

[20] Kuga D Ushida K Mii S Enomoto A Asai N Nagino M. Tyrosine Phosphorylation of an Actin-Binding Protein Girdin Specifically Marks Tuft Cells in Human and Mouse Gut. J Histochem Cytochem (2017) 65:347–66. doi: 10.1369/0022155417702586 PMC5625852 28375676

[21] Schütz B Ruppert A-L Strobel O Lazarus M Urade Y Büchler MW. Distribution Pattern and Molecular Signature of Cholinergic Tuft Cells in Human Gastro-Intestinal and Pancreatic-Biliary Tract. Sci Rep (2019) 9:17466. doi: 10.1038/s41598-019-53997-3 31767912 PMC6877571

[22] Haber AL Biton M Rogel N Herbst RH Shekhar K Smillie C. A Single-Cell Survey of the Small Intestinal Epithelium. Nature (2017) 551:333–9. doi: 10.1038/nature24489 PMC6022292 29144463

[23] Kjærgaard S Jensen TSR Feddersen UR Bindslev N Grunddal KV Poulsen SS. Decreased Number of Colonic Tuft Cells in Quiescent Ulcerative Colitis Patients. Eur J Gastroenterol Hepatol (2021) 33:817–24. doi: 10.1097/MEG.0000000000001959 PMC8083166 33079783

[24] Beumer J Artegiani B Post Y Reimann F Gribble F Nguyen TN. Enteroendocrine Cells Switch Hormone Expression Along the Crypt-to-Villus BMP Signalling Gradient. Nat Cell Biol (2018) 20:909–16. doi: 10.1038/s41556-018-0143-y PMC6276989 30038251

[25] Gehart H van Es JH Hamer K Beumer J Kretzschmar K Dekkers JF. Identification of Enteroendocrine Regulators by Real-Time Single-Cell Differentiation Mapping. Cell (2019) 176:1158–73. doi: 10.1016/j.cell.2018.12.029 30712869

[26] Grunddal KV Tonack S Egerod KL Thompson JJ Petersen N Engelstoft MS. Adhesion Receptor Adgrg2/Gpr64 Is in the GI-Tract Selectively Expressed in Mature Intestinal Tuft Cells. Mol Metab (2021) 101231. doi: 10.1016/j.molmet.2021.101231 PMC8105302 33831593

[27] Nakanishi Y Seno H Fukuoka A Ueo T Yamaga Y Maruno T. Dclk1 Distinguishes Between Tumor and Normal Stem Cells in the Intestine. Nat Genet (2013) 45:98–103. doi: 10.1038/ng.2481 23202126

[28] Westphalen CB Asfaha S Hayakawa Y Takemoto Y Lukin DJ Nuber AH. Long-Lived Intestinal Tuft Cells Serve as Colon Cancer-Initiating Cells. J Clin Invest (2014) 124:1283–95. doi: 10.1172/JCI73434 PMC3934168 24487592

[29] Yui S Azzolin L Maimets M Pedersen MT Fordham RP Hansen SL. YAP/TAZ-Dependent Reprogramming of Colonic Epithelium Links ECM Remodeling to Tissue Regeneration. Cell Stem Cell (2018) 22:35–49. doi: 10.1016/j.stem.2017.11.001 29249464 PMC5766831

[30] Bjerknes M Khandanpour C Möröy T Fujiyama T Hoshino M Klisch TJ. Origin of the Brush Cell Lineage in the Mouse Intestinal Epithelium. Dev Biol (2012) 362:194–218. doi: 10.1016/j.ydbio.2011.12.009 22185794 PMC6050067

[31] Yamashita J Ohmoto M Yamaguchi T Matsumoto I Hirota J. Skn-1a/Pou2f3 Functions as a Master Regulator to Generate Trpm5-Expressing Chemosensory Cells in Mice. PloS One (2017) 12:e0189340. doi: 10.1371/journal.pone.0189340 29216297 PMC5720759

[32] Gerbe F Sidot E Smyth DJ Ohmoto M Matsumoto I Dardalhon V. Intestinal Epithelial Tuft Cells Initiate Type 2 Mucosal Immunity to Helminth Parasites. Nature (2016) 529:226–30. doi: 10.1038/nature16527 PMC7614903 26762460

[33] Matsumoto I Ohmoto M Narukawa M Yoshihara Y Abe K. Skn-1a (Pou2f3) Specifies Taste Receptor Cell Lineage. Nat Neurosci (2011) 14:685–7. doi: 10.1038/nn.2820 PMC3390744 21572433

[34] O’Leary CE Schneider C Locksley RM. Tuft Cells-Systemically Dispersed Sensory Epithelia Integrating Immune and Neural Circuitry. Annu Rev Immunol (2019) 37:47–72. doi: 10.1146/annurev-immunol-042718-041505 30379593 PMC8352721

[35] Bornstein C Nevo S Giladi A Kadouri N Pouzolles M Gerbe F. Single-Cell Mapping of the Thymic Stroma Identifies IL-25-Producing Tuft Epithelial Cells. Nature (2018) 559:622–6. doi: 10.1038/s41586-018-0346-1 30022162

[36] Montoro DT Haber AL Biton M Vinarsky V Lin B Birket SE. A Revised Airway Epithelial Hierarchy Includes CFTR-Expressing Ionocytes. Nature (2018) 560:319–24. doi: 10.1038/s41586-018-0393-7 PMC6295155 30069044

[37] Plasschaert LW Žilionis R Choo-Wing R Savova V Knehr J Roma G. A Single-Cell Atlas of the Airway Epithelium Reveals the CFTR-Rich Pulmonary Ionocyte. Nature (2018) 560:377–81. doi: 10.1038/s41586-018-0394-6 PMC6108322 30069046

[38] von Moltke J Ji M Liang H-E Locksley RM. Tuft-Cell-Derived IL-25 Regulates an Intestinal ILC2-Epithelial Response Circuit. Nature (2016) 529:221–5. doi: 10.1038/nature16161 PMC4830391 26675736

[39] Herring CA Banerjee A McKinley ET Simmons AJ Ping J Roland JT. Unsupervised Trajectory Analysis of Single-Cell RNA-Seq and Imaging Data Reveals Alternative Tuft Cell Origins in the Gut. Cell Syst (2018) 6:37–51. doi: 10.1016/j.cels.2017.10.012 29153838 PMC5799016

[40] Banerjee A Herring CA Chen B Kim H Simmons AJ Southard-Smith AN. Succinate Produced by Intestinal Microbes Promotes Specification of Tuft Cells to Suppress Ileal Inflammation. Gastroenterology (2020) 159:2101–15. doi: 10.1053/j.gastro.2020.08.029 PMC7725941 32828819

[41] Schumacher MA Hsieh JJ Liu CY Appel KL Waddell A Almohazey D. Sprouty2 Limits Intestinal Tuft and Goblet Cell Numbers Through GSK3β-Mediated Restriction of Epithelial IL-33. Nat Commun (2021) 12:836. doi: 10.1038/s41467-021-21113-7 33547321 PMC7864916

[42] Aigbologa J Connolly M Buckley JM O’Malley D. Mucosal Tuft Cell Density Is Increased in Diarrhea-Predominant Irritable Bowel Syndrome Colonic Biopsies. Front Psychiatry (2020) 11:436. doi: 10.3389/fpsyt.2020.00436 32477197 PMC7242613

[43] O’Donnell AM Nakamura H Puri P. Tuft Cells: A New Player in Hirschsprung’s Disease. Eur J Pediatr Surg (2020) 30:59–63. doi: 10.1055/s-0039-1700549 31707728

[44] Leppänen J Helminen O Huhta H Kauppila JH Miinalainen I Ronkainen VP. Doublecortin-Like Kinase 1-Positive Enterocyte – a New Cell Type in Human Intestine. APMIS (2016) 124:958–65. doi: 10.1111/apm.12599 27677532

[45] McKinley ET Sui Y Al-Kofahi Y Millis BA Tyska MJ Roland JT. Optimized Multiplex Immunofluorescence Single-Cell Analysis Reveals Tuft Cell Heterogeneity. JCI Insight (2017) 2:e93487. doi: 10.1172/jci.insight.93487 PMC5453701 28570279

[46] Billipp TE Nadjsombati MS von Moltke J. Tuning Tuft Cells: New Ligands and Effector Functions Reveal Tissue-Specific Function. Curr Opin Immunol (2021) 68:98–106. doi: 10.1016/j.coi.2020.09.006 33166855 PMC7925335

[47] Huh WJ Te RJ Asai M Kaji I. Distribution of Duodenal Tuft Cells Is Altered in Pediatric Patients With Acute and Chronic Enteropathy. BioMed Res (2020) 41:113–8. doi: 10.2220/biomedres.41.113 PMC10037909 32307399

[48] Ahmad R Dalziel JE. G Protein-Coupled Receptors in Taste Physiology and Pharmacology. Front Pharmacol (2020) 11:587664. doi: 10.3389/fphar.2020.587664 33390961 PMC7774309

[49] Howitt MR Cao YG Gologorsky MB Li JA Haber AL Biton M. The Taste Receptor TAS1R3 Regulates Small Intestinal Tuft Cell Homeostasis. ImmunoHorizons (2020) 4:23–32. doi: 10.4049/immunohorizons.1900099 31980480 PMC7197368

[50] Howitt MR Lavoie S Michaud M Blum AM Tran SV Weinstock JV. Tuft Cells, Taste-Chemosensory Cells, Orchestrate Parasite Type 2 Immunity in the Gut. Science (2016) 351:1329–33. doi: 10.1126/science.aaf1648 PMC5528851 26847546

[51] Luo XC Chen ZH Xue JB Zhao DX Lu C Li YH. Infection by the Parasitic Helminth Trichinella Spiralis Activates a Tas2r-Mediated Signaling Pathway in Intestinal Tuft Cells. Proc Natl Acad Sci USA (2019) 116:5564–9. doi: 10.1073/pnas.1812901116 PMC6431192 30819885

[52] Widmayer P Partsch V Pospiech J Kusumakshi S Boehm U Breer H. Distinct Cell Types With the Bitter Receptor Tas2r126 in Different Compartments of the Stomach. Front Physiol (2020) 11:32. doi: 10.3389/fphys.2020.00032 32116750 PMC7019106

[53] Hass N Schwarzenbacher K Breer H. T1R3 Is Expressed in Brush Cells and Ghrelin-Producing Cells of Murine Stomach. Cell Tissue Res (2010) 339:493–504. doi: 10.1007/s00441-009-0907-6 20063013

[54] Nadjsombati MS McGinty JW Lyons-Cohen MR Jaffe JB DiPeso L Schneider C. Detection of Succinate by Intestinal Tuft Cells Triggers a Type 2 Innate Immune Circuit. Immunity (2018) 49:33–41. doi: 10.1016/j.immuni.2018.06.016 30021144 PMC6084797

[55] Bezençon C Fürholz A Raymond F Mansourian R Métairon S Le Coutre J. Murine Intestinal Cells Expressing Trpm5 Are Mostly Brush Cells and Express Markers of Neuronal and Inflammatory Cells. J Comp Neurol (2008) 509:514–25. doi: 10.1002/cne.21768 18537122

[56] Arora P Andersen D Moll JM Danneskiold-Samsøe NB Xu L Zhou B. Small Intestinal Tuft Cell Activity Associates With Energy Metabolism in Diet-Induced Obesity. Front Immunol (2021) 12:1745. doi: 10.3389/fimmu.2021.629391 PMC8195285 34122403

[57] Elmentaite R Kumasaka N Roberts K Fleming A Dann E King HW. Cells of the Human Intestinal Tract Mapped Across Space and Time. Nature (2021) 597:250–5. doi: 10.1101/2021.04.07.438755 PMC8426186 34497389

[58] Prandi S Voigt A Meyerhof W Behrens M. Expression Profiling of Tas2r Genes Reveals a Complex Pattern Along the Mouse GI Tract and the Presence of Tas2r131 in a Subset of Intestinal Paneth Cells. Cell Mol Life Sci (2018) 75:49–65. doi: 10.1007/s00018-017-2621-y 28801754 PMC11105753

[59] Rozengurt N Wu SV Chen MC Huang C Sternini C Rozengurt E. Colocalization of the α-Subunit of Gustducin With PYY and GLP-1 in L Cells of Human Colon. Am J Physiol - Gastrointest Liver Physiol (2006) 291:792–802. doi: 10.1152/ajpgi.00074.2006 16728727

[60] Jeruzal-Świątecka J Fendler W Pietruszewska W. Clinical Role of Extraoral Bitter Taste Receptors. Int J Mol Sci (2020) 21:1–23. doi: 10.3390/ijms21145156 PMC7404188 32708215

[61] Wu SV Rozengurt N Yang M Young SH Sinnett-Smith J Rozengurt E. Expression of Bitter Taste Receptors of the T2R Family in the Gastrointestinal Tract and Enteroendocrine STC-1 Cells. Proc Natl Acad Sci USA (2002) 99:2392–7. doi: 10.1073/pnas.042617699 PMC122375 11854532

[62] Rozengurt E. Taste Receptors in the Gastrointestinal Tract. I. Bitter Taste Receptors and α-Gustducin in the Mammalian Gut. Am J Physiol - Gastrointest Liver Physiol (2006) 291:171–7. doi: 10.1152/ajpgi.00073.2006 16710053

[63] Latorre R Huynh J Mazzoni M Gupta A Bonora E Clavenzani P. Expression of the Bitter Taste Receptor, T2R38, in Enteroendocrine Cells of the Colonic Mucosa of Overweight/Obese vs. Lean Subjects. PloS One (2016) 11:e0147468. doi: 10.1371/journal.pone.0147468 26866366 PMC4750998

[64] Wang Q Liszt KI Deloose E Canovai E Thijs T Farré R. Obesity Alters Adrenergic and Chemosensory Signaling Pathways That Regulate Ghrelin Secretion in the Human Gut. FASEB J (2019) 33:4907–20. doi: 10.1096/fj.201801661RR 30629462

[65] Gu F Liu X Liang J Chen J Chen F Li F. Bitter Taste Receptor Mtas2r105 Is Expressed in Small Intestinal Villus and Crypts. Biochem Biophys Res Commun (2015) 463:934–41. doi: 10.1016/j.bbrc.2015.06.038 26071358

[66] Yamazaki T Takahashi C Taniguchi Y Narukawa M Misaka T Ano Y. Bitter Taste Receptor Activation by Hop-Derived Bitter Components Induces Gastrointestinal Hormone Production in Enteroendocrine Cells. Biochem Biophys Res Commun (2020) 533:704–9. doi: 10.1016/j.bbrc.2020.10.099 33160623

[67] Liu S Lu S Xu R Atzberger A Günther S Wettschureck N. Members of Bitter Taste Receptor Cluster Tas2r143/Tas2r135/Tas2r126 Are Expressed in the Epithelium of Murine Airways and Other Non-Gustatory Tissues. Front Physiol (2017) 8:1–17. doi: 10.3389/fphys.2017.00849 29163195 PMC5670347

[68] Bezençon C le Coutre J Damak S. Taste-Signaling Proteins Are Coexpressed in Solitary Intestinal Epithelial Cells. Chem Senses (2007) 32:41–9. doi: 10.1093/chemse/bjl034 17030556

[69] Höfer D Püschel B Drenckhahn D. Taste Receptor-Like Cells in the Rat Gut Identified by Expression of α-Gustducin. Proc Natl Acad Sci USA (1996) 93:6631–4. doi: 10.1073/pnas.93.13.6631 PMC39077 8692869

[70] Hass N Schwarzenbacher K Breer H. A Cluster of Gustducin-Expressing Cells in the Mouse Stomach Associated With Two Distinct Populations of Enteroendocrine Cells. Histochem Cell Biol (2007) 128:457–71. doi: 10.1007/s00418-007-0325-3 17874119

[71] Drurey C Lindholm HT Coakley G Poveda MC Löser S Doolan R. Intestinal Epithelial Tuft Cell Induction Is Negated by a Murine Helminth and Its Secreted Products. J Exp Med (2022) 219:e20211140. doi: 10.1084/jem.20211140 34779829 PMC8597987

[72] Schütz B Jurastow I Bader S Ringer C Engelhardt JV Chubanov V. Chemical Coding and Chemosensory Properties of Cholinergic Brush Cells in the Mouse Gastrointestinal and Biliary Tract. Front Physiol (2015) 6:87. doi: 10.3389/fphys.2015.00087 25852573 PMC4371653

[73] Schneider C O’Leary CE von Moltke J Liang HE Ang QY Turnbaugh PJ. A Metabolite-Triggered Tuft Cell-ILC2 Circuit Drives Small Intestinal Remodeling. Cell (2018) 174:271–84. doi: 10.1016/j.cell.2018.05.014 PMC6046262 29887373

[74] Krantis A. GABA in the Mammalian Enteric Nervous System. News Physiol Sci (2000) 15:284–90. doi: 10.1152/physiologyonline.2000.15.6.284 11390928

[75] Aggarwal S Ahuja V Paul J. Attenuated Gabaergic Signaling in Intestinal Epithelium Contributes to Pathogenesis of Ulcerative Colitis. Dig Dis Sci (2017) 62:2768–79. doi: 10.1007/s10620-017-4662-3 28667430

[76] Ma X Sun Q Sun X Chen D Wei C Yu X. Activation of GABAA Receptors in Colon Epithelium Exacerbates Acute Colitis. Front Immunol (2018) 9:987. doi: 10.3389/fimmu.2018.00987 29867964 PMC5949344

[77] McGinty JW Ting HA Billipp TE Nadjsombati MS Khan DM Barrett NA. Tuft-Cell-Derived Leukotrienes Drive Rapid Anti-Helminth Immunity in the Small Intestine But Are Dispensable for Anti-Protist Immunity. Immunity (2020) 52:528–41. doi: 10.1016/j.immuni.2020.02.005 PMC7469474 32160525

[78] Lei W Ren W Ohmoto M Urban JF Matsumoto I Margolskee RF. Activation of Intestinal Tuft Cell-Expressed Sucnr1 Triggers Type 2 Immunity in the Mouse Small Intestine. Proc Natl Acad Sci USA (2018) 115:5552–7. doi: 10.1073/pnas.1720758115 PMC6003470 29735652

[79] Chu C Parkhurst CN Zhang W Zhou L Yano H Arifuzzaman M. The Chat-Acetylcholine Pathway Promotes Group 2 Innate Lymphoid Cell Responses and Anti-Helminth Immunity. Sci Immunol (2021) 6:eabe3218. doi: 10.1126/sciimmunol.abe3218 33674322 PMC8577047

[80] Roberts LB Schnoeller C Berkachy R Darby M Pillaye J Oudhoff MJ. Acetylcholine Production by Group 2 Innate Lymphoid Cells Promotes Mucosal Immunity to Helminths. Sci Immunol (2021) 6:eabd0359. doi: 10.1126/sciimmunol.abd0359 33674321

[81] Terashima A Watarai H Inoue S Sekine E Nakagawa R Hase K. A Novel Subset of Mouse NKT Cells Bearing the IL-17 Receptor B Responds to IL-25 and Contributes to Airway Hyperreactivity. J Exp Med (2008) 205:2727–33. doi: 10.1084/jem.20080698 PMC2585837 19015310

[82] Neill DR Mckenzie ANJ. Nuocytes and Beyond: New Insights Into Helminth Expulsion. Trends Parasitol (2019) 27:214–21. doi: 10.1016/j.pt.2011.01.001 21292555

[83] Wang D Dubois RN. Eicosanoids and Cancer. Nat Rev Cancer (2010) 10:181–93. doi: 10.1038/nrc2809 PMC2898136 20168319

[84] Oyesola OO Shanahan MT Kanke M Mooney BM Webb LM Smita S. PGD2 and CRTH2 Counteract Type 2 Cytokine–Elicited Intestinal Epithelial Responses During Helminth Infection. J Exp Med (2021) 218:e20202178. doi: 10.1084/jem.20202178 34283207 PMC8294949

[85] Dai L King DW Perera DS Lubowski DZ Burcher E Liu L. Inverse Expression of Prostaglandin E2-Related Enzymes Highlights Differences Between Diverticulitis and Inflammatory Bowel Disease. Dig Dis Sci (2015) 60:1236–46. doi: 10.1007/s10620-014-3478-7 25666316

[86] Hendel J Nielsen OH. Expression of Cyclooxygenase-2 mRNA in Active Inflammatory Bowel Disease. Am J Gastroenterol (1997) 92:1170–3. 9219792

[87] Montrose DC Nakanishi M Murphy RC Zarini S McAleer JP Vella AT. The Role of PGE2 in Intestinal Inflammation and Tumorigenesis. Prostaglandins Other Lipid Mediat (2015) 116–117:26–36. doi: 10.1016/j.prostaglandins.2014.10.002 PMC4385488 25460828

[88] Fung C Howitt MR. A Tuft Act to Follow: Leukotrienes Take the Stage in Anti-Worm Immunity. Immunity (2020) 52:426–8. doi: 10.1016/j.immuni.2020.02.011 32187512

[89] O’Leary CE Feng X Cortez VS Locksley RM Schneider C. Interrogating the Small Intestine Tuft Cell–ILC2 Circuit Using *In Vivo* Manipulations. Curr Protoc (2021) 1:e77. doi: 10.1002/cpz1.77 33740294 PMC8082719

[90] Fallon PG Ballantyne SJ Mangan NE Barlow JL Dasvarma A Hewett DR. Identification of an Interleukin (IL)-25-Dependent Cell Population That Provides IL-4, IL-5, and IL-13 at the Onset of Helminth Expulsion. J Exp Med (2006) 203:1105–16. doi: 10.1084/jem.20051615 PMC2118283 16606668

[91] Middelhoff M Nienhüser H Valenti G Maurer HC Hayakawa Y Takahashi R. Prox1-Positive Cells Monitor and Sustain the Murine Intestinal Epithelial Cholinergic Niche. Nat Commun (2020) 11:111. doi: 10.1038/s41467-019-13850-7 31913277 PMC6949263

[92] Saunders CJ Christensen M Finger TE Tizzano M. Cholinergic Neurotransmission Links Solitary Chemosensory Cells to Nasal Inflammation. Proc Natl Acad Sci USA (2014) 111:6075–80. doi: 10.1073/pnas.1402251111 PMC4000837 24711432

[93] Hollenhorst MI Jurastow I Nandigama R Appenzeller S Li L Vogel J. Tracheal Brush Cells Release Acetylcholine in Response to Bitter Tastants for Paracrine and Autocrine Signaling. FASEB J (2020) 34:316–32. doi: 10.1096/fj.201901314RR 31914675

[94] Perniss A Liu S Boonen B Keshavarz M Ruppert AL Timm T. Chemosensory Cell-Derived Acetylcholine Drives Tracheal Mucociliary Clearance in Response to Virulence-Associated Formyl Peptides. Immunity (2020) 52:683–99. doi: 10.1016/j.immuni.2020.03.005 32294408

[95] Jönsson M Norrgård Ö Forsgren S. Presence of a Marked Nonneuronal Cholinergic System in Human Colon: Study of Normal Colon and Colon in Ulcerative Colitis. Inflammation Bowel Dis (2007) 13:1347–56. doi: 10.1002/ibd.20224 17663429

[96] Damm MMB Jensen TSR Mahmood B Lundh M Poulsen SS Bindslev N. Acetylcholine-Related Proteins in Non-Neoplastic Appearing Colonic Mucosa From Patients With Colorectal Neoplasia. Mol Carcinog (2017) 56:2223–33. doi: 10.1002/mc.22675 28544328

[97] Bueno L Fioramonti J. Action of Opiates on Gastrointestinal Function. Baillieres Clin Gastroenterol (1988) 2:123–39. doi: 10.1016/0950-3528(88)90024-3 2838107

[98] Kokrashvili Z Rodriguez D Yevshayeva V Zhou H Margolskee RF Mosinger B. Release of Endogenous Opioids From Duodenal Enteroendocrine Cells Requires Trpm5. Gastroenterology (2009) 137:598–606. doi: 10.1053/j.gastro.2009.02.070 19272386 PMC2717179

[99] Saenz SA Taylor BC Artis D. Welcome to the Neighborhood: Epithelial Cell-Derived Cytokines License Innate and Adaptive Immune Responses at Mucosal Sites. Immunol Rev (2008) 226:172–90. doi: 10.1111/j.1600-065X.2008.00713.x PMC2683382 19161424

[100] Zeuthen LH Fink LN Frokiaer H. Epithelial Cells Prime the Immune Response to an Array of Gut-Derived Commensals Towards a Tolerogenic Phenotype Through Distinct Actions of Thymic Stromal Lymphopoietin and Transforming Growth Factor-β. Immunology (2008) 123:197–208. doi: 10.1111/j.1365-2567.2007.02687.x 17655740 PMC2433297

[101] Allakhverdi Z Comeau MR Jessup HK Yoon BRP Brewer A Chartier S. Thymic Stromal Lymphopoietin Is Released by Human Epithelial Cells in Response to Microbes, Trauma, or Inflammation and Potently Activates Mast Cells. J Exp Med (2007) 204:253–8. doi: 10.1084/jem.20062211 PMC2118732 17242164

[102] Park JH Jeong DY Peyrin-biroulet L Eisenhut M Shin J. Insight Into the Role of TSLP in Inflammatory Bowel Diseases. Autoimmun Rev (2017) 16:55–63. doi: 10.1016/j.autrev.2016.09.014 27697608

[103] Heylen M Ruyssers NE Gielis EM Vanhomwegen E Pelckmans PA Moreels TG. Of Worms, Mice and Man: An Overview of Experimental and Clinical Helminth-Based Therapy for Inflammatory Bowel Disease. Pharmacol Ther (2014) 143:153–67. doi: 10.1016/j.pharmthera.2014.02.011 24603369

[104] Schölmerich J Fellermann K Seibold FW Rogler G Langhorst J Howaldt S. A Randomised, Double-Blind, Placebo-Controlled Trial of Trichuris Suis Ova in Active Crohn’s Disease. J Crohn’s Colitis (2017) 11:390–9. doi: 10.1093/ecco-jcc/jjw184 PMC5881737 27707789

[105] Huang X Zeng L Chen F Zhu J Zhu M. Trichuris Suis Ova Therapy in Inflammatory Bowel Disease. Medicine (2018) 97:e12087. doi: 10.1097/MD.0000000000012087 30142867 PMC6113037

[106] Sandborn WJ Elliott DE Weinstock J Summers RW Landry-Wheeler A Silver N. Randomised Clinical Trial: The Safety and Tolerability of Trichuris Suis Ova in Patients With Crohn’s Disease. Aliment Pharmacol Ther (2013) 38:255–63. doi: 10.1111/apt.12366 23730956

[107] Summers RW Elliott DE Qadir K Urban JF Thompson R Weinstock JV. Trichuris Suis Seems to be Safe and Possibly Effective in the Treatment of Inflammatory Bowel Disease. Am J Gastroenterol (2003) 98:2034–41. doi: 10.1111/j.1572-0241.2003.07660.x 14499784

[108] Borkow G Leng Q Weisman Z Stein M Galai N Kalinkovich A. Chronic Immune Activation Associated With Intestinal Helminth Infections Results in Impaired Signal Transduction and Anergy. J Clin Invest (2000) 106:1053–60. doi: 10.1172/JCI10182 PMC314342 11032865

[109] Desai P Janova H White JP Reynoso GV Hickman HD Baldridge MT. Enteric Helminth Coinfection Enhances Host Susceptibility to Neurotropic Flaviviruses *via* a Tuft Cell-IL-4 Receptor Signaling Axis. Cell (2021) 184:1214–31. doi: 10.1016/j.cell.2021.01.051 PMC7962748 33636133

[110] Wilen CB Lee S Hsieh LL Orchard RC Desai C Hykes BL. Tropism for Tuft Cells Determines Immune Promotion of Norovirus Pathogenesis. Science (2018) 360:204–8. doi: 10.1126/science.aar3799 PMC6039974 29650672

[111] Teunis PFM Sukhrie FHA Vennema H Bogerman J Beersma MFC Koopmans MPG. Shedding of Norovirus in Symptomatic and Asymptomatic Infections. Epidemiol Infect (2015) 143:1710–7. doi: 10.1017/S095026881400274X PMC9507237 25336060

[112] Li Q Ma L Shen S Guo Y Cao Q Cai X. Intestinal Dysbacteriosis-Induced IL-25 Promotes Development of HCC *via* Alternative Activation of Macrophages in Tumor Microenvironment. J Exp Clin Cancer Res (2019) 38:303. doi: 10.1186/s13046-019-1271-3 31296243 PMC6625119

[113] Buonomo EL Madan R Pramoonjago P Li L Okusa MD Petri WA. Role of Interleukin 23 Signaling in Clostridium Difficile Colitis. J Infect Dis (2013) 208:917–20. doi: 10.1093/infdis/jit277 PMC3749013 23776194

[114] Cowardin CA Petri WA. Host Recognition of Clostridium Difficile and the Innate Immune Response. Anaerobe (2014) 30:205–9. doi: 10.1016/j.anaerobe.2014.08.014 PMC4258135 25223264

[115] Kleinschek MA Owyang AM Joyce-Shaikh B Langrish CL Chen Y Gorman DM. IL-25 Regulates Th17 Function in Autoimmune Inflammation. J Exp Med (2007) 204:161–70. doi: 10.1084/jem.20061738 PMC2118427 17200411

[116] Buonomo EL Cowardin CA Wilson MG Saleh MM Pramoonjago P Petri WA. Microbiota-Regulated IL-25 Increases Eosinophil Number to Provide Protection During Clostridium Difficile Infection. Cell Rep (2016) 16:432–43. doi: 10.1016/j.celrep.2016.06.007 PMC4945404 27346351

[117] Kobayashi T Siegmund B Le Berre C Wei SC Ferrante M Shen B. Ulcerative Colitis. Nat Rev Dis Prim (2020) 6:74. doi: 10.1038/s41572-020-0205-x 32913180

[118] Roda G Chien Ng S Kotze PG Argollo M Panaccione R Spinelli A. Crohn’s Disease. Nat Rev Dis Prim (2020) 6:22. doi: 10.1038/s41572-020-0156-2 32242028

[119] Chang JT. Pathophysiology of Inflammatory Bowel Diseases. N Engl J Med (2020) 383:2652–64. doi: 10.1056/NEJMra2002697 33382932

[120] Strober W Fuss IJ. Proinflammatory Cytokines in the Pathogenesis of Inflammatory Bowel Diseases. Gastroenterology (2011) 140:1756–67. doi: 10.1053/j.gastro.2011.02.016 PMC3773507 21530742

[121] Su J Chen T Ji X-Y Liu C Yadav PK Wu R. IL-25 Downregulates Th1/Th17 Immune Response in an IL-10 – Dependent Manner in Inflammatory Bowel Disease. Inflammation Bowel Dis (2013) 19:720–8. doi: 10.1097/MIB.0b013e3182802a76 23429464

[122] Liu Y Shao Z Shangguan G Bie Q Zhang B. Biological Properties and the Role of IL-25 in Disease Pathogenesis. J Immunol Res (2018) 2018:6519465. doi: 10.1155/2018/6519465 30345318 PMC6174801

[123] Kunze B Middelhoff M Maurer HC Agibalova T Anand A Bührer A-M. Notch Signaling Drives Development of Barrett’s Metaplasia From Dclk1-Positive Epithelial Tuft Cells in the Murine Gastric Mucosa. Sci Rep (2021) 11:1–13. doi: 10.1038/s41598-021-84011-4 33627749 PMC7904766

[124] Roulis M Kaklamanos A Schernthanner M Bielecki P Zhao J Kaffe E. Paracrine Orchestration of Intestinal Tumorigenesis by a Mesenchymal Niche. Nature (2020) 580:524–9. doi: 10.1038/s41586-020-2166-3 PMC7490650 32322056

[125] Wang D DuBois RN. Fibroblasts Fuel Intestinal Tumorigenesis. Cell Res (2020) 30:635–6. doi: 10.1038/s41422-020-0340-7 PMC7395122 32494022

[126] Petersen CH Mahmood B Badsted C Dahlby T Rasmussen HB Hansen MB. Possible Predisposition for Colorectal Carcinogenesis Due to Altered Gene Expressions in Normal Appearing Mucosa From Patients With Colorectal Neoplasia. BMC Cancer (2019) 19:1–13. doi: 10.1186/s12885-019-5833-8 31253108 PMC6599319

[127] Fearon ER Vogelstein B. A Genetic Model for Colorectal Tumorigenesis. Cell (1990) 61:759–67. doi: 10.1016/0092-8674(90)90186-I 2188735

